# Reliance of Scientific Publication on Citation Management Software: The New Generation

**DOI:** 10.5681/joddd.2012.032

**Published:** 2012-12-11

**Authors:** Vahid Rakhshan

**Affiliations:** Department of Dental Anatomy and Morphology, Dental Branch, Islamic Azad University, Tehran, Iran

A great deal of critical and creative thinking is needed for authoring a good paper. Nevertheless, there is a simple task during manuscript preparation, namely reference listing, which, although needs no intelligence, it can seem extremely tedious and time-consuming to authors.

Apart from the impact of computers on many aspects of research, they can be a dream tool for dealing with repetitious, dull, yet precise tasks,^1^ including bibliography management. Computer software can fetch accurate citation data from the official websites, insert them into a database without errors, and allow for their batch-editing as required. One such commercially available application is “End-Note” and a free alternative to that is “Zotero”, a worthwhile new-generation bibliography manage-ment software.^2-5^

Zotero (available at http://www.zotero.org) is a free open-source cross-platform citation manager that can be used to “collect, organize, cite, and share” research resources.^2-5^ It easily captures avail-able bibliographic information with minimal mouse clicks as single citations or bundles of multiple references while browsing a website containing an article or relevant information. There is a large and still growing number of websites supported by Zotero including Web of Science, PubMed, PubMed Central, ScienceDirect, Google Scholar, Google Books, Wikipedia, Amazon, and many other websites of renowned publishers which hold publication data such as DOI, ISBN, or PMID.^2-5^

Unlike other programs of its kind, Zotero is ex tremely fast in fetching data, compatible with virtu-ally all operating systems, and very simple to learn. Zotero only requires Mozilla Firefox (a free web browser) and supports free and commercial word processor programs,^2-5^ many of which are not supported by other similar software. Moreover, its ability to export bibliographic data into EndNote (RIS) format is another advantage. A researcher who is familiar with EndNote can still utilize Zotero to capture citations, and then feed them into EndNote for further procedures. Mendeley (available at http://www.mendeley.com) has recently developed a similar feature; however, using Zotero seems to be much more convenient. A brief comparison is given in Table 1, with detailed comparisons available on the Internet (e.g., the comparison on Wikipedia).

**Table 1:** A brief comparison of four citation manager programs

Item	EndNote X5	Mendeley 1.6	Zotero 3	RefWorks
Supporting platforms	Windows / Mac	Cross-platform	Cross-platform	Windows / Mac
Software type	Standalone	Standalone	Standalone / Browser	Online
Price	US$299.9	Free^a^	Free^b^	US$100/year
Fetch citations while surfing	—	•	•	—
Merging fields in a document	•	—	—	—
Manual citation input	•	•	•	•
Style editor GUI	•	—	—	•
Number of bibliographic styles	+5000	+1100^c^	+2700	+3200
Merging duplicate references	—	•	•	—

Data obtained from manufacturer websites or personal testing. GUI: Graphical user interface. ^a^Online storage for Mendeley costs about US$60/year. ^b^Zotero synchronization is free for storages less than 100 megabytes and is US$20/year for one gigabyte. ^c^Mendeley citation styles are acquired from Zotero repository.

When it comes to managing the citations in a word processor program, however, EndNote has the upper hand. Unlike almost all other citation managers, EndNote can automatically merge citation fields when being put together, or is able to insert new citations right into the field of an existing citation. These features are extremely handy when working with a lot of references, as users can simply put two or more citations together, and let EndNote convert them to a single citation field, a feature not available in the current version of Zotero, but requested from developers by the author. Also, EndNote can be programmed to use one of different journal title abbreviations, while Zotero is limited to be fed only by the citation file. Besides, EndNote has a graphical user interface for editing citation styles, while the current version of Zotero requires the editing of CSL style file using a text editor, a process that, despite being simple, is not as convenient as the EndNote’s approach.

However, these features and many other suggestions have been requested by community members, and some are under development right now. The open-source nature of Zotero and its highly responsive community make quick enhancements possible. For example, corrections/modifications made to the Vancouver style by the author were rapidly incorporated in the online style repository and were then swiftly enhanced further by other community members.

Zotero is not all about referencing. Similar to Mendeley, it provides features such as catching or importing attachments like PDF or HTML files, annotating, highlighting, indexing the captured files, searching within them, and also synchronizing and sharing them on a free online 100 megabyte space.^2-5^ In addition, it can display the studies on a graphical timeline, based on the months of publication, a nice feature ignored in other applications. A unique, handy feature of Zotero is that it can automatically detect proxied resources such as publisher websites subscribed by the user’s institution. Once a user clicks the link of an article located on a subscribed domain using the Firefox browser, Zotero automatically redirects to the same preferred journal website page, however, through the off-campus proxy channel. Therefore, in order to be able to obtain full articles, the user does not need to access the websites of desired journals only from the control panel or search box of the off-campus service, which is not usually as user-friendly or robust as renowned search engines like Google. This brilliant feature can save the researcher considerable time and allow them to still benefit from convenient interface of powerful search engines while still having full access to their off-campus service.

The convenient, rapid data capturing potential of Zotero and its compatibility with many other powerful applications of its kind can synergy to accelerate the procedure of scientific writing, which seems vital to academics in rush to publish rather than perish.
